# Mirabegron Versus Tamsulosin in Reducing Double-J Stent-Related Symptoms: A Prospective Comparative Study

**DOI:** 10.7759/cureus.91925

**Published:** 2025-09-09

**Authors:** Santosh R Patil, Kiran K Negi, Siddanagouda B Patil, Vinay S Kundargi

**Affiliations:** 1 Urology, BLDE (Deemed to be University) Shri B. M. Patil Medical College, Hospital and Research Centre, Vijayapura, IND

**Keywords:** mirabegron, stent-related symptoms, tamsulosin, ureteral stent, ureteral stent symptom questionnaire (ussq)

## Abstract

Introduction

Double-J (DJ) ureteral stents are widely used in endourology to relieve upper tract obstruction and ensure adequate postoperative drainage. However, stent-related symptoms (SRS), including lower urinary tract symptoms (LUTS), flank pain, haematuria, and discomfort, significantly impair patient quality of life. Pharmacologic interventions, such as alpha-blockers and beta-3 adrenergic agonists, have been proposed to mitigate SRS. This study aimed to compare the efficacy and safety of mirabegron versus tamsulosin in reducing DJ SRS.

Materials and methods

This prospective observational study was conducted at Shri B. M. Patil Medical College, Vijayapura, India (IEC No: BLDE (DU)/IEC/997/2022-23), between July 2023 and July 2024. A total of 130 adult patients with indwelling ureteral stents following endourological procedures were enrolled and allocated into two groups: Group A received tamsulosin 0.4 mg once daily, and Group B received mirabegron 50 mg once daily, continued until stent removal. Symptom assessment was performed at two and three weeks using the validated Ureteral Stent Symptom Questionnaire (USSQ). Safety profiles were also documented.

Results

Both groups demonstrated progressive symptom improvement over three weeks. Mirabegron showed significantly lower urinary symptom scores (12.1 vs. 15.6 at two weeks; 9.4 vs. 13.2 at three weeks, p < 0.001) and body pain scores (4.5 vs. 6.8 at two weeks; 3.1 vs. 5.4 at three weeks, p < 0.001). Improvements in general health, sexual function, and work performance favoured mirabegron, but were not statistically significant. Mirabegron exhibited a favourable safety profile, with fewer minor adverse events.

Conclusions

Mirabegron 50 mg once daily provides superior relief of urinary symptoms and pain compared to tamsulosin 0.4 mg once daily, with good tolerability and improved overall quality of life in patients with indwelling DJ ureteral stents.

## Introduction

Ureteral stent insertion remains a cornerstone in urological practice to relieve upper urinary tract obstruction and ensure adequate drainage following procedures such as ureteroscopy (URS), percutaneous nephrolithotomy (PCNL), and retrograde intrarenal surgery (RIRS) [1]. By maintaining urine flow and preventing complications like severe flank pain, fever, and sepsis, these stents significantly enhance postoperative outcomes [1].

Despite their benefits, double-J (DJ) stents are frequently associated with bothersome adverse effects. Joshi et al. and Lee et al. reported that 50%-80% of patients experience stent-related side effects, collectively termed stent-related symptoms (SRS) [2,3]. These symptoms encompass a spectrum of lower urinary tract symptoms (LUTS), including urinary frequency (60%), urgency (60%), dysuria (40%), haematuria (54%), and flank or suprapubic pain in up to 80% of patients [4-6]. To quantify stent-related morbidity, the Ureteral Stent Symptom Questionnaire (USSQ) serves as the validated gold standard tool, enabling objective assessment and inter-study comparisons [7,8].

Multiple strategies have been explored to alleviate SRS, including modifications in stent design, surface coatings, and pharmacologic interventions. However, the optimal management approach remains uncertain. Alpha-1 adrenergic receptor antagonists (alpha-blockers) have demonstrated efficacy in mitigating LUTS associated with benign prostatic hyperplasia by reducing bladder outlet resistance and alleviating symptoms like frequency, urgency, and suprapubic pain [9]. Conversely, anti-muscarinic and beta-3 adrenergic receptor agonists, such as mirabegron, target stent-induced overactive bladder (OAB) by suppressing involuntary detrusor contractions triggered by trigonal irritation [10].

Given the high prevalence of SRS and its impact on patient comfort, evaluating pharmacologic approaches for symptom relief is clinically significant. Therefore, this study aims to assess the efficacy of mirabegron 50 mg once daily versus tamsulosin 0.4 mg once daily in reducing ureteral DJ SRS.

## Materials and methods

Study design

This was a prospective observational study conducted at Shri B. M. Patil Medical College, Vijayapura, India, from July 2023 to July 2024, following approval from the Institutional Ethics Committee (IEC No: BLDE (DU)/IEC/997/2022-23).

Patient recruitment

A total of 130 patients who underwent endourological procedures requiring placement of a ureteral DJ stent were included. Eligible patients were 18-70 years old and developed LUTS related to the indwelling stent. Exclusion criteria were: pre-existing OAB, chronic pelvic pain syndrome, active urinary tract infection, pregnancy or lactation, uncontrolled hypertension, prior ureteric reconstruction surgery, urinary tract malignancy, bilateral stenting, or long-term stents on regular exchange protocols.

Sample size calculation

Sample size was estimated using the formula for comparing two independent means: $n = \frac{(Z_{\alpha/2} + Z_{\beta})^2 \times 2\sigma^2}{\Delta^2}$.

Considering a mean difference (Δ) of 0.3 in LUTS severity score between groups [11], a standard deviation (σ) of 0.59, a 95% confidence level (Zα/2 = 1.96), and 80% power (Zβ = 0.84), the required sample size was 60 per group. To account for potential dropouts, 65 patients were recruited in each group (total = 130).

Data collection and intervention

Patients were prospectively allocated into two groups using a consecutive sampling approach, ensuring that all eligible patients were included until the required sample size was reached. Group A (n = 65) received tamsulosin 0.4 mg once daily, and Group B (n = 65) received mirabegron 50 mg once daily, continued until stent removal. Symptom evaluation was performed using the USSQ, which assesses urinary symptoms, body pain, general health, sexual health, work performance, and additional health problems. The USSQ was administered two weeks after stent placement and again at three weeks, during stent removal. Demographic and clinical data were systematically recorded.

Statistical analysis

Quantitative data were expressed as mean ± standard deviation and compared using Student’s t-test or ANOVA, as appropriate. Categorical variables were analysed using the Chi-square test. All hypothesis tests were two-tailed. A p-value < 0.05 was considered statistically significant.

## Results

A total of 130 patients were analysed, with 65 in each group. No patients were lost to follow-up. Group A (tamsulosin) included 51 males (78.46%) and 14 females (21.54%), while Group B (mirabegron) included 48 males (73.85%) and 17 females (26.15%). The mean age was 49.07 ± 12.31 years in Group A and 45.77 ± 10.76 years in Group B. There were no significant differences in baseline demographics or stone characteristics between the groups (Table 1).

**Table 1:** Baseline Demographic and Clinical Characteristics of Patients This table summarizes the baseline characteristics of patients allocated to the tamsulosin and mirabegron groups. Continuous variables, such as age, body mass index (BMI), and stone size, are presented as mean ± standard deviation (SD) and compared using the independent sample t-test. Categorical variables, such as gender, stone site, stone side, and type of procedure, are presented as frequencies and percentages, with comparisons performed using the Chi-square (χ²) test. The two groups were comparable across all measured variables, with no statistically significant differences observed (p > 0.05). PCNL, percutaneous nephrolithotomy; URS, ureteroscopy; RIRS, retrograde intrarenal surgery

Variable		Tamsulosin (n = 65)	Mirabegron (n = 65)	Statistical value (χ²/t)	p-value
Age (years)	Mean ± SD	49.07 ± 12.31	45.77 ± 10.76	t = 1.43	0.23
Gender	Male	51 (78.46%)	48 (73.85%)	χ² = 0.24	0.62
	Female	14 (21.54%)	17 (26.15%)		
BMI	kg/m²	24.55 ± 1.54	23.85 ± 1.89	t = 1.73	0.09
Stone Site	Calyx	3 (4.61%)	5 (7.69%)	χ² = 4.02	0.40
	Pelvis	11 (16.92%)	15 (23.08%)		
	Upper ureter	19 (29.23%)	13 (20.00%)		
	Mid ureter	9 (13.84%)	12 (18.46%)		
	Lower ureter	23 (35.38%)	20 (30.77%)		
Stone Side	Left	22 (33.84%)	16 (24.61%)	χ² = 1.14	0.28
	Right	43 (66.15%)	49 (75.38%)		
Stone Size	Mm	8.6 ± 2.5	7.9 ± 3.5	t = 1.27	0.21
Procedure Type	PCNL	17 (26.15%)	20 (30.77%)	χ² = 0.53	0.76
	URS	39 (60.00%)	38 (58.46%)		
	RIRS	9 (13.85%)	7 (10.77%)		

**Figure 1:**
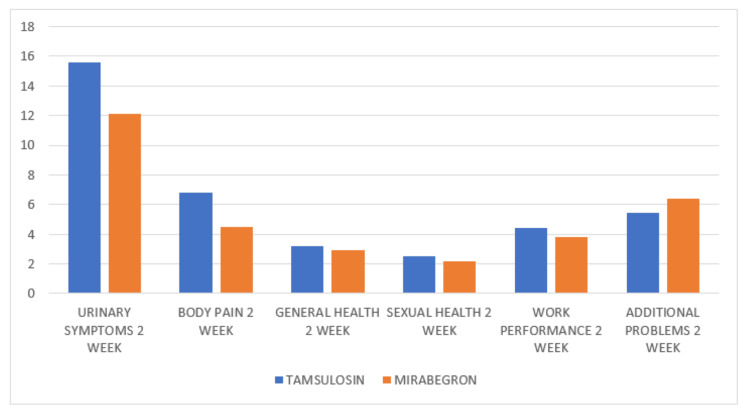
Bar Chart Showing USSQ Score (Tamsulosin vs Mirabegron) Two Weeks Post-operative This bar chart illustrates the mean scores (y-axis) across six USSQ domains - urinary symptoms, body pain, general health, sexual health, work performance, and additional health problems - at two weeks post-procedure. Patients treated with mirabegron consistently reported lower urinary symptoms and body pain scores compared to those receiving tamsulosin, indicating better tolerability. Other domains, including general health, sexual health, work performance, and additional problems, did not show notable differences between groups. Lower scores represent fewer stent-related symptoms and improved quality of life. USSQ, Ureteral Stent Symptom Questionnaire

**Figure 2:**
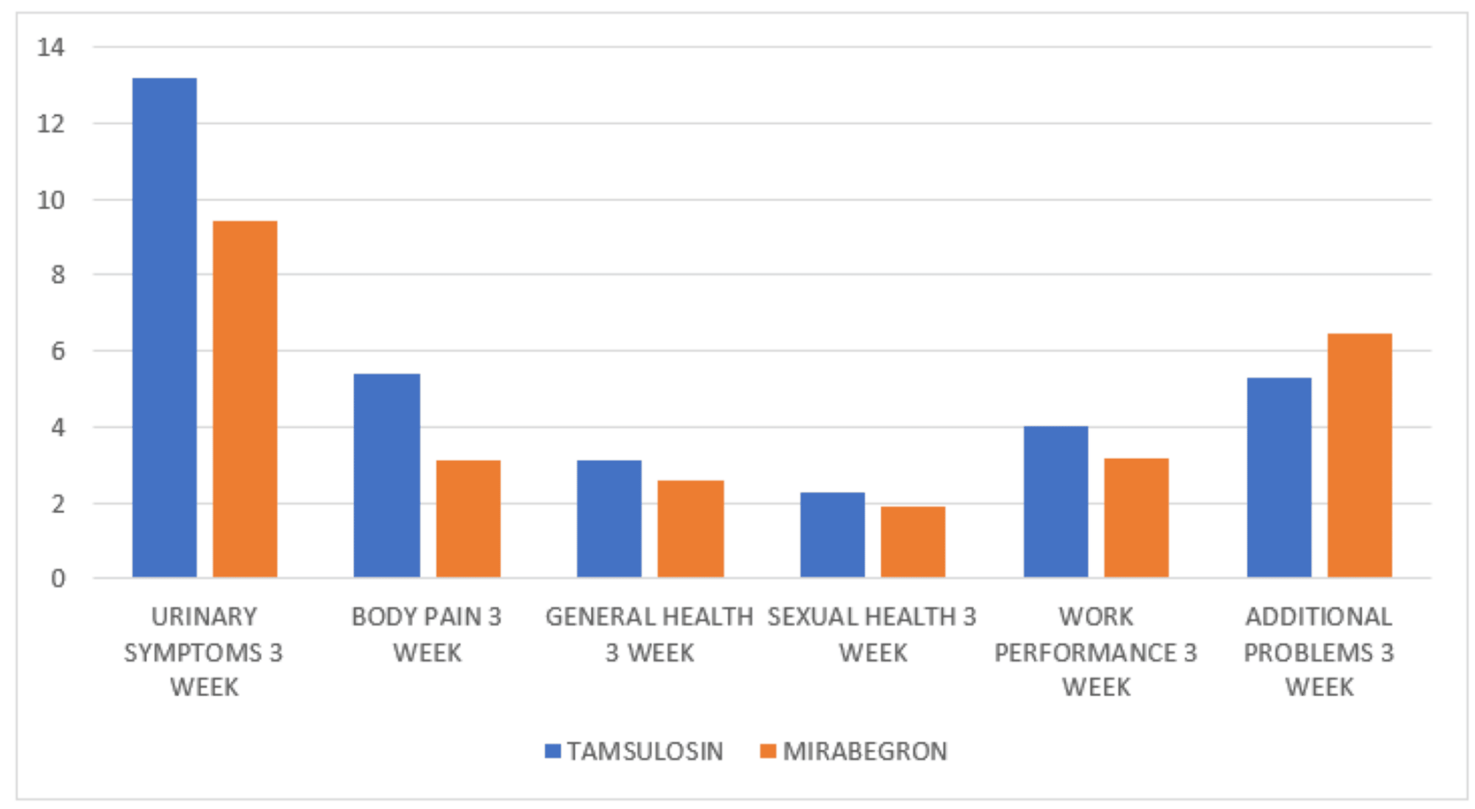
Bar Chart Showing USSQ Score (Tamsulosin vs Mirabegron) Three Weeks Post-operative This bar chart shows the mean USSQ domain scores at three weeks post-procedure for both treatment groups. Mirabegron maintained lower urinary symptom and body pain scores than tamsulosin, reinforcing its superiority in symptom reduction over time. Scores for general health, sexual health, and work performance were comparable between groups, while additional health problem scores remained slightly higher in the mirabegron group. Lower scores indicate fewer symptoms and better stent tolerability. USSQ, Ureteral Stent Symptom Questionnaire

At two weeks, the mirabegron group demonstrated significantly lower urinary symptom scores (12.1 vs 15.6; p = 0.002), with a further reduction by week 3 (9.4 vs 13.2; p < 0.001). Body pain scores were also lower in the mirabegron group at two weeks (4.5 vs 6.8; p = 0.001) and three weeks (3.1 vs 5.4; p < 0.001). Other USSQ domains, including general health, sexual health, work performance, and additional health problems, showed numerical improvements in the mirabegron group but were not statistically significant (Table 2; Figures 1-2).

**Table 2:** USSQ Scores at Two and Three Weeks This table presents the mean ± standard deviation (SD) scores for each domain of the USSQ at two follow-up intervals (two weeks and three weeks), in patients receiving either tamsulosin or mirabegron. Independent sample t-tests were used to compare continuous variables between groups. Lower scores in each domain indicate fewer symptoms and better tolerability. At both time points, mirabegron demonstrated significantly lower scores for urinary symptoms and body pain, compared to tamsulosin (p < 0.05), suggesting superior symptom relief. No statistically significant differences were observed between the groups for general health, sexual health, work performance, or additional health problem domains (p > 0.05). USSQ, Ureteral Stent Symptom Questionnaire

USSQ Domain	Timepoint	Tamsulosin (Mean ± SD)	Mirabegron (Mean ± SD)	t-value	p-value	Bonferroni-corrected p-value
Urinary Symptoms	2 weeks	15.6 ± 3.2	12.1 ± 2.8	3.19	0.002	0.012
	3 weeks	13.2 ± 2.6	9.4 ± 2.1	4.64	<0.001	0.006
Body Pain	2 weeks	6.8 ± 1.9	4.5 ± 1.6	3.41	0.001	0.003
	3 weeks	5.4 ± 1.8	3.1 ± 1.3	4.38	<0.001	0.001
General Health	2 weeks	3.2 ± 1.0	2.9 ± 0.9	1.26	0.21	1.26
	3 weeks	3.1 ± 1.1	2.6 ± 0.8	1.79	0.07	0.42
Sexual Health	2 weeks	2.5 ± 0.8	2.2 ± 0.7	1.45	0.15	0.90
	3 weeks	2.3 ± 0.9	1.9 ± 0.6	1.78	0.08	0.48
Work Performance	2 weeks	4.4 ± 1.5	3.8 ± 1.3	1.62	0.11	0.66
	3 weeks	4.0 ± 1.4	3.2 ± 1.2	1.98	0.05	0.30
Additional Health Problem	2 weeks	5.43 ± 1.57	6.37 ± 2.11	1.57	0.12	0.72
	3 weeks	5.29 ± 2.08	6.47 ± 2.36	1.65	0.10	0.60

Mirabegron demonstrated a more favourable safety profile compared to tamsulosin. Only one case of palpitations was reported with mirabegron. In the tamsulosin group, dry mouth (1, 1.54%), dizziness (2, 3.08%), palpitations (1, 1.54%), and headache (1, 1.54%) were observed. No cases of urinary retention or hypertension were noted in either group (Table 3).

**Table 3:** Comparison of Adverse Effects in Both Groups This table summarizes the incidence of common adverse effects in the two treatment groups. Data are presented as the number of cases with corresponding percentages. Group comparisons were performed using the Chi-square test, where applicable. Overall, adverse events were infrequent and mild. Dry mouth, dizziness, and headache were reported only in the tamsulosin group, though differences were not statistically significant (p > 0.05). Palpitations were reported equally in both groups, while no cases of urinary retention or hypertension were observed in either group.

Adverse effect	Tamsulosin (n = 65)	Mirabegron (n = 65)	Chi-square value	p-value
Dry mouth	1 (1.54%)	0 (0%)	1.01	0.31
Dizziness	2 (3.08%)	0 (0%)	2.03	0.15
Palpitations	1 (1.54%)	1 (1.54%)	-	-
Headache	1 (1.54%)	0 (0%)	1.01	0.31
Urinary retention	0 (0%)	0 (0%)	-	-
Hypertension	0 (0%)	0 (0%)	-	-

## Discussion

Indwelling ureteric stents are indispensable in urological practice but are frequently associated with SRS, which significantly impair patients’ quality of life. Joshi et al. reported a high prevalence of urinary dysfunction and stent-related pain in patients with indwelling ureteric stents [5]. These symptoms are primarily attributed to bladder mucosal irritation, triggering lower ureteral and bladder spasms [11]. Attempts to reduce SRS have included stent length and diameter optimization, positioning adjustments to prevent the distal loop from crossing the bladder midline, and material modifications [12]. However, conflicting evidence persists, with some studies reporting no significant impact of stent dimensions on SRS severity [13].

Technological efforts to develop coated and biomaterial stents have been made to reduce biofilm formation and mucosal irritation, but no current stent design has successfully eliminated SRS [14,15]. Pharmacotherapy remains the cornerstone of SRS management, including analgesics, anticholinergics, and alpha-blockers [15-17]. Alpha-1 blockers, such as tamsulosin, act by reducing smooth muscle tone in the ureter, bladder trigone, and prostatic urethra, thereby alleviating spasms [15,18,19]. Beta-3 adrenergic agonists, like mirabegron, relax bladder and ureteral smooth muscle, reduce ureteral spasms, and may decrease pelvic pressure and vesicoureteral reflux [14,15]. Given the overlap between SRS and OAB symptoms, mirabegron has gained interest as a potential therapy [15].

Previous studies have demonstrated mixed outcomes for mirabegron in SRS management. Tae et al. showed that mirabegron significantly reduced pain and overall discomfort but did not improve USSQ urinary or general health scores [15]. Yavuz et al. found that mirabegron reduced the need for analgesics but failed to achieve statistically significant urinary symptom improvement [14]. Shalaby et al. reported enhanced SRS relief with combined anticholinergic and alpha-blocker therapy but raised tolerability concerns [20]. In contrast, Cinar et al. observed that mirabegron monotherapy significantly reduced SRS in a retrospective study [21].

Our findings align with and expand upon these reports. Both tamsulosin and mirabegron improved USSQ scores during the three-week indwelling period. However, mirabegron demonstrated superior relief in urinary symptoms and body pain scores at both two and three weeks (p < 0.001), with trends toward improved general health, sexual function, and work performance domains, although these did not reach statistical significance. These results are consistent with a study by Sahin et al. [22], supporting mirabegron’s efficacy and favourable tolerability profile for stent-related discomfort.

The study’s strengths include its prospective design, the use of a validated and standardized USSQ questionnaire, and complete follow-up without patient attrition, ensuring reliable assessment of symptom progression. The direct head-to-head comparison of mirabegron and tamsulosin monotherapy also provides clinically actionable insights into the pharmacologic management of SRS.

This study has several limitations. It was conducted at a single centre with a modest sample size, which may limit the generalizability of the results. Long-term outcomes beyond three weeks were not assessed, and the study did not evaluate combination therapies, which could potentially offer greater symptom relief. Additionally, variations in stent design and material were not stratified, which may have contributed to symptom variability. Finally, patients were allocated using a non-randomized, consecutive sampling approach, which introduces potential selection bias and may have influenced the comparability of the groups despite similar baseline characteristics.

## Conclusions

Mirabegron 50 mg once daily demonstrated superior efficacy over tamsulosin 0.4 mg once daily in alleviating ureteral SRS, with statistically significant improvements in the urinary symptom and body pain domains of the USSQ. While general health, sexual function, and work performance showed favourable trends with mirabegron, these did not reach statistical significance. Its favourable safety profile and enhanced symptom relief highlight mirabegron as an effective, well-tolerated option for improving the quality of life in patients with indwelling ureteral stents.
